# An old test for new neurons: refining the Morris water maze to study the functional relevance of adult hippocampal neurogenesis

**DOI:** 10.3389/fnins.2013.00063

**Published:** 2013-05-03

**Authors:** Alexander Garthe, Gerd Kempermann

**Affiliations:** ^1^German Center for Neurodegenerative Diseases (DZNE)Dresden, Germany; ^2^CRTD – DFG Research Center for Regenerative Therapies Dresden, Technische Universität DresdenDresden, Germany

**Keywords:** hippocampus, adult neurogenesis, water maze, spatial learning, pattern separation, dentate gyrus, CA3, flexibility

## Abstract

The Morris water maze represents the de-facto standard for testing hippocampal function in laboratory rodents. In the field of adult hippocampal neurogenesis, however, using this paradigm to assess the functional relevance of the new neurons yielded surprisingly inconsistent results. While some authors found aspects of water maze performance to be linked to adult neurogenesis, others obtained different results or could not demonstrate any effect of manipulating adult neurogenesis. In this review we discuss evidence that the large diversity of protocols and setups used is an important aspect in interpreting the differences in the results that have been obtained. Even simple parameters such as pool size, number, and configuration of visual landmarks, or number of trials can become highly relevant for getting the new neurons involved at all. Sets of parameters are often chosen with implicit or explicit concepts in mind and these might lead to different views on the function of adult-generated neurons. We propose that the classical parameters usually used to measure spatial learning performance in the water maze might not be particularly well-suited to sensitively and specifically detect the supposedly highly specific functional changes elicited by the experimental modulation of adult hippocampal neurogenesis. As adult neurogenesis is supposed to affect specific aspects of information processing only in the hippocampus, any claim for a functional relevance of the new neurons has to be based on hippocampus-specific parameters. We also placed a special emphasis on the fact that the dentate gyrus (DG) facilitates the differentiation between contexts as opposed to just differentiating places. In conclusion, while the Morris water maze has proven to be one of the most effective testing paradigms to assess hippocampus-dependent spatial learning, new and more specific questions ask for new parameters. Therefore, the full potential of the water maze task remains to be tapped.

Adult hippocampal neurogenesis deserves much of its attention due to the fact that it adds new functional neurons to a brain structure that is of particular importance for learning and memory processes (Kempermann, 2012). While the relevance of adult neurogenesis for pattern separation that was predicted by theoretical models has been demonstrated by a couple of studies, the functional contribution of the adult-born granule cells in the context of more complex spatial learning tasks remained unclear. Since a large number of studies has shown that many aspects of spatial learning depend on an intact and fully functional hippocampus and given that for at least some species the generation of new hippocampal neurons in the adult brain appears to provide an advantage in evolution (Kempermann, 2012), it is somewhat surprising that neither a significant reduction nor an increase of adult neurogenesis were consistently found to result in a strong and specific phenotype in the water maze task. Thus, adult neurogenesis does not seem to be beneficial for hippocampal and brain function *per se*, but rather contributes to highly specific functional aspects of spatial learning that become apparent only when certain task demands are faced.

A complex spatial learning task like the Morris water maze explicitly refers to challenges routinely experienced under naturalistic conditions and, consequently, task performance does not depend exclusively on the hippocampus (Morris, 1984). A substantial body of lesion experiments has revealed that to varying degrees key parameters of spatial water maze learning are dependent on hippocampal function, while many other aspects are not (Redish, 1999).

Because adult-born new neurons are exclusively found in the dentate gyrus (DG) and become integrated only into local hippocampal circuitry, their functional contribution is intimately related to the truly hippocampus-dependent aspects of spatial learning. Measuring latencies or path lengths to find the hidden platform still represent the most widely used read-out parameters of water maze experiments, although they do not allow to address specifically the hippocampal contribution to learning performance. This problem to an even higher degree also applies to adult neurogenesis, as the new neurons are supposed to participate in specific hippocampal functions only rather than influencing information processing in general, i.e., by randomly adding new, highly excitable neurons. Furthermore, there is a wide range of protocols and setups being used, each one posing specific challenges on the animals' spatial learning abilities. While some experimental designs appear to address functional parameters related to adult neurogenesis quite effectively, others largely fail to do so yielding false-negative results (Leuner et al., 2006; Zhao et al., 2008; Aimone et al., 2010; Leuner and Gould, 2010).

In this review, we will look at factors that potentially affect whether and to what extent adult neurogenesis becomes involved in learning the water maze, followed by a discussion of alternative read-out parameters that reflect the functional contribution of the new neurons more specifically. We begin with a general overview of how information is processed by the hippocampus, placing a specific emphasis on the supposed functions of the DG and hippocampal subarea CA3, both being of particular relevance in the contexts of adult neurogenesis and spatial navigation.

## Adult hippocampal neurogenesis

Neuronal precursors arise from glia-like stem cells in the subgranular zone (SGZ) of the DG and finally differentiate into excitatory granule cells, the principal cell type of the DG (Kempermann, 2012). Expanding the pool of cells that potentially can become recruited and thus exit the cell cycle takes place on the stage of precursors. Most of the new cells are removed by apoptosis, but 10–50% survive and become functionally integrated in already existing networks of the DG, where they can persist for month or even lifelong. It has also been shown that proliferation of precursor cells is modulated by physical activity whereas living in a stimulus-rich enriched environment increases survival of the new neurons (Kempermann et al., 1997; Van Praag et al., 1999).

Adult neurogenesis encompasses a maturation process over multiple stages, characterized by a unique pattern of morphology, marker expression, and responsiveness to electrical stimulation and sensitivity to regulation by physical or cognitive stimuli (Kronenberg et al., 2003; Kempermann et al., 2004; Steiner et al., 2008; Tronel et al., 2010). Thus, the integration of the new neurons is modulated by the actually expressed behavior, adjusting the development of new hippocampal granule cells to the acute functional needs.

In some sense there is thus not just a single type of neurons available for functional integration which can be used or not. Rather the distinct maturational stages with their different properties and different responsiveness to regulatory stimuli provide a considerable variety of adult-born neuronal cells that might fulfill different functions. Theoretical models have pointed toward different functions of newborn neurons depending on their respective maturation stages (Aimone et al., 2006, 2009; Deng et al., 2010). Overall, adult neurogenesis appears to provide a rather new form of plasticity and it has been hypothesized, that the addition of new neurons over lifetime helps to keep hippocampal information processing efficient. This is summarized in the neurogenic reserve hypothesis (Kempermann, 2008).

The following section provides an overview of hippocampal information processing with a special emphasis on the DG. Implications of theoretical models of the functional relevance of adult neurogenesis that appear to be especially relevant in the context of spatial learning are discussed, followed by mentioning an important electrophysiological finding, that provides an important hint what kinds of experimental setups and protocol might be especially effective for addressing the functional contribution of the new neurons.

## Functional roles of adult neurogenesis – implications from theoretical models

The hippocampus is one of the most extensively studied structures in the mammalian brain (Anderson et al., 2006). Being part of the well-conserved “allocortex” and thus made up by just three as compared to the six cortical layers, it shows an appealingly clear backbone structure, the so called “trisynaptic circuit” (Amaral and Witter, 1995; Anderson et al., 2006). Information from virtually all higher sensory cortical areas is converging on the entorhinal cortex (EC) and via the perforant path conveyed to the first relay station of the circuit, the DG. From here information is passed to CA3 (second relay station), further relayed to CA1 (third station), and transmitted back to the EC via the subiculum. Somewhat contrary to the high amount of information from cortical areas that is converging in the EC and subsequently has to be processed by the hippocampal circuitry, the DG shows only an extreme sparse pattern of activation. This has been interpreted as the DG representing a competitive learning network that implements a strong compression and orthogonalization of information intended to be stored in CA3 (Rolls, 2010).

It has also been found that information is conveyed directly from the EC to CA3 (Anderson et al., 2006). Because input relayed via the DG to CA3 is delayed compared to contents using the direct connection, it has been hypothesized that adult neurogenesis facilitates the fine-tuning of how the DG makes orthogonalization and storage in CA3 more efficient (Wu and Leung, 1998).

**Figure 1:**
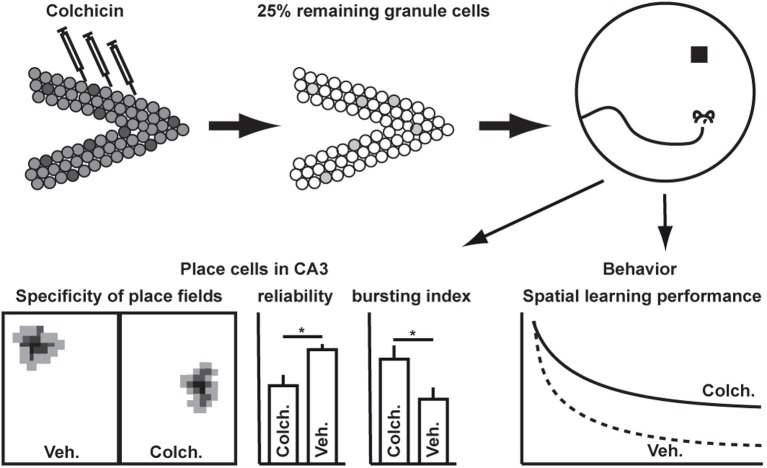
**Spatial specificity of place cells in CA3 does not depend on input from the dentate gyrus [as shown by McNaughton et al. (1989)].** Ablation of dentate granule cells causes spatial learning deficits on the behavioral level and increases the bursting probability of pyramidal cells in CA3. ^*^p < 0.01.

The highly recurrent network structure of area CA3 strongly implies that it serves as a temporary autoassociative memory (Rolls, 1996). In spatial tasks, spiking of CA3 pyramidal cells is highly correlated with the animals' specific position in the arena (Nadel and O'Keefe, 1978). Place cells appear upon introduction to a new environment (Hill, 1978; Shapiro et al., 1997) and provide the neuronal basis for the concept of cognitive maps in the hippocampus. Intriguingly, it was found that spatial specificity of place cells in CA3 does not depend on input from the DG (McNaughton et al., 1989). After ablation of more than two-third of all granule cells in the DG, CA3 pyramidal neurons still showed selective place fields (Figure 1). The study revealed, that the mossy fibers provide information regarding the actual context and thus input from the DG does not seem to be relevant in determining where but when hippocampal place cells fire. While the spatial specificity of place cells in the hippocampus basically represents a built-in property of hippocampal circuitry, the input from the DG allows to link a specific location to the actual behavioral context, i.e., approaching the actual goal from the present location in the most efficient way. Although hippocampal learning is generally taken as an equivalent for spatial learning, this finding together with the fact that sensory information of nearly all modalities is fed into the hippocampus underscores the necessity to keep a much broader role of the DG in dealing with contexts in mind. The specific relevance of these findings in the context of adult neurogenesis is discussed below.

Considering the amount of information fed into the hippocampus via the EC, the storage capacity of the CA3 network turns out to be rather limited and therefore, the main function generally assigned to the DG is the compression and orthogonalization of activity patterns to make them suitable for storage in CA3 (Marr, 1971; Treves et al., 2008). That pattern separation is indeed a key function of the DG was elegantly demonstrated by Hunsaker et al. (Hunsaker and Kesner, 2008).

It has been proposed that without efficient pattern separation representations related to new unknown contexts that have to be stored in CA3 would interfere with older, already encoded ones, thereby preventing the effective storage and retrieval of highly similar but nonetheless different representations (Aimone et al., 2006, 2009; Wiskott et al., 2006; Appleby and Wiskott, 2009; Appleby et al., 2011). Because adult hippocampal neurogenesis adds new neurons to the DG, it is assumed that the function of adult neurogenesis is to prevent such “catastrophic interference” by keeping pattern separation in the DG effective while facing a high variety of different but partly overlapping contexts—that is: input-patterns—over life time. Because the adult-born new neurons become indistinguishable from the already existing dentate granule cells and are just added in comparable small numbers, adult neurogenesis likely is not adding qualitatively new functions to hippocampal information processing.

## The morris water maze task

The Morris water maze was introduced by Richard Morris more than 30 years ago and represents the canonical task to assess spatial learning in laboratory rodents (Morris et al., 1982). The water maze consists of a rather simple setup: a circular pool is filled with water that has been made opaque by adding a white pigment (or even milk powder). Animals are required to escape from the water onto a small platform hidden just beneath the water surface. The location of the platform can only be encoded relative to distal visual landmarks surrounding the water maze pool and thus using spatial memory. Stable visual cues can be either provided using a curtain around the pool or by the objects that are placed at different locations in the testing room.

The swim path of an animal is recorded using a camera on the ceiling connected to a computer running a tracking-software. Based on the time-tagged xy-coordinates most available software packages calculate parameters describing different aspects of spatial learning performance, such as latency to reach the hidden platform, path length, time in quadrants, number of goal crossings, velocity, or floating times.

A considerable variety of protocols has been developed but these can be usually subsumed into one of two general categories, the reference memory version and the delayed matching to place (DMP) procedure (Anderson et al., 2006). Independent of the specific protocol used, a water maze task consists of multiple trials, each trial typically lasting a maximum fixed time between 60 and 120 s. The animals are introduced into the water facing the pool wall at defined starting positions.

For the reference memory protocol the platform position remains stable across trials and days. Acquisition until latency to find the hidden platform has reached a stable minimum typically takes 8–10 days, although this depends on pool size, number of available visual landmarks, age of the animals tested as well as on the specific species and strain that were used. Subvariants of the reference memory protocol either use a new starting position for each single trial or keep a starting position constant within each day. Following acquisition, learning performance is assessed using a probe trial in which the platform is removed from the pool, and the animal is allowed to freely swim around for 60 s. Successful spatial learning is indicated by a clear preference for the goal quadrant as well as an above chance probability to cross the former goal location. Thus, the reference memory version addresses the formation and further modification of an cognitive map that contains not only information on the general spatial layout of the testing arena but also allows to recognize specific locations that can be remembered as part of a route and thus further used in subsequent trials.

Some protocols include a platform reversal after animals have learned to navigate to a given goal position. As previously mentioned, the spatial relationships between specific spatial contexts and the platform position change upon goal reversal and thus successful navigation has to be relearned. As discussed later, reversal learning appears to represent a functional challenge where adult neurogenesis is especially relevant for separating contexts effectively.

In turn, the DMP protocol places emphasis on one-trial learning as animals have to encode the goal location within a single trial, followed by one or more trials after a certain inter-trial-interval (ITI). By varying the ITI the experimenter can reassemble procedures similar to the DMP task usually applied to assess recognition memory. The essential difference to the reference memory version lies in the fact that the tested animals are not allowed to acquire and consolidate spatial knowledge for many trials and over multiple days. Therefore, the DMP protocol is considered to assess spatial working memory.

Importantly, performance in the water maze task does not exclusively rely on the hippocampus. A substantial body of lesion experiments, pharmacological, and genetic manipulations revealed a role in mediating different aspects of spatial learning for numerous brain structures beside the hippocampus, i.e., nucleus accumbens (movement directions) and caudate nucleus (place-reward association), lateral mammillary and thalamic nuclei (head direction), posterior parietal cortex (local panoramic view system), subiculum, EC (grid cells), and superior colliculus (orientation toward specific cues) [for an extensive review see Redish (1999)].

**Figure 2:**
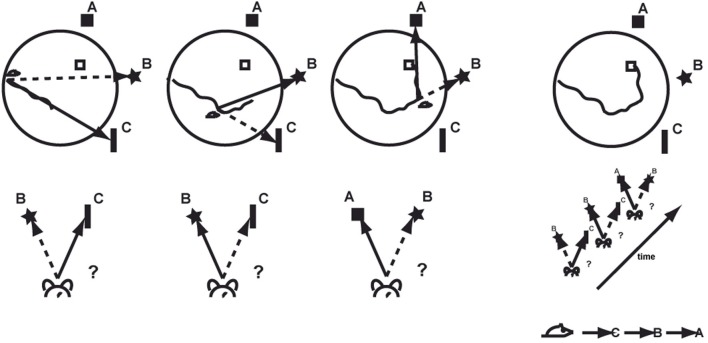
**Associating specific places or spatial contexts with moving directions allows learning effective routes to find the hidden goal.** A, B, and C represent different stable visual landmarks.

Successful navigation to the hidden platform depends on different behavioral abilities and their coordinated interaction. Recognizing a specific spatial context means to identify a specific panoramic view previously seen and remembered (Figure 2). During task acquisition, stable and predictive landmarks have to be selected and remembered from all available visual cues. Introducing the animal the first time to the pool establishes a spatial frame of reference based on prominent landmarks and their geometric configurations. When the animal is moving, it has to keep track on its heading direction and an integrated representation of the path traveled, a process requiring vector arithmetic. Over time, animals do experience and possibly remember certain routes or at least fragments of routes that allow reaching the goal efficiently. Once a location has been recognized as being part of an already known route it can be selected over the currently used one depending on the actual goal position. As an overarching aspect, the goal's position has to be remembered and associated with the reward of being rescued. It is presumed that the role of the hippocampus is to localize the animal on the cognitive map, and that it facilitates efficient navigation through a host of other anatomical and functional structures.

**Figure 3:**
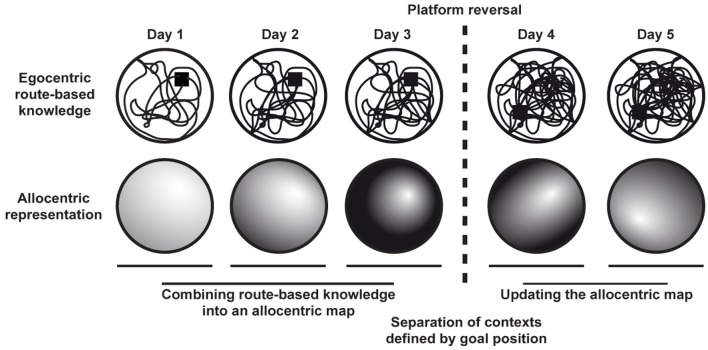
**Formation and updating of allocentric cognitive maps in a task with changing goal positions.** Egocentric route-based knowledge from repeated trials becomes integrated into an allocentric cognitive map. Contexts are defined by different goal positions. Accordingly, upon platform reversal the allocentric cognitive map has to be updated to allow efficient navigation in the new context.

In essence, learning the water maze is based on the successful integration of egocentric, route-based knowledge into an allocentric representation that is independent of recognizing specific contexts as parts of already known routes (Figure 3). While the mere existence of an allocentric cognitive map is an all-or-nothing phenomenon, the specificity with which important places such as goals are encoded in such a cognitive map clearly depends on the underlying egocentric experiences and thus on the known contexts. Thus, the more detailed the egocentric, route-based coverage of an environment the greater can be the spatial specificity how the hidden platform gets encoded in the resulting allocentric map. As a consequence, an animal's behavior depends on both egocentric and allocentric knowledge, the contribution of the latter increasing with repeated trials.

## Factors affecting experimental evidence for a role of adult neurogenesis in water maze learning

The water maze task has been used in a considerable number of studies addressing the functional relevance of adult neurogenesis. While some studies revealed a functional role for adult neurogenesis, the overall evidence for a specific contribution of the new neurons appears to be inconsistent. However, a meta-analysis of the water maze studies revealed three domains of the task that were repeatedly shown being specifically affected by an absence of new hippocampal neurons: task acquisition, probe trial performance, and re-acquisition after moving the platform to another location (reversal). While most studies found a lower probe trial performance, an impaired task acquisition is less consistently reported, and only a few studies observed the reversal phenotype upon moving the platform to a new position.

The ablation methods used in early studies on the functional relevance of adult neurogenesis caused severe side effects that compromised the animals' overall learning abilities (Shors et al., 2002). Also, it was not known that for the water maze task the new neurons must be of a certain age to become recruited into hippocampal networks (Kee et al., 2007). But even using sophisticated and highly specific ablation methods as well as experimental schedules targeting the new neurons at specific ages did not consistently yield at least one of the aforementioned phenotypes.

However, taking into account the respective species, protocols, setups, and parameters used as well as the age of the animals tested, suggest those parameters as important factors determining whether and partly what phenotype can be found. Studies using rats reported a reduced preference for the goal quadrant after suppression of adult neurogenesis (Snyder et al., 2005; Winocur et al., 2006; Jessberger et al., 2009). Setups testing mice without curtains around the pool or at least using highly complex patterns instead of simple geometric figures reliably found an impaired task acquisition (Dupret et al., 2008; Zhang et al., 2008; Garthe et al., 2009; Martinez-Canabal et al., 2013). Using such a setup along with a protocol including a new stable platform position revealed the reversal phenotype (Zhang et al., 2008; Garthe et al., 2009). Finally, a recent study provided evidence that the functional consequences of a reduced adult neurogenesis might be more relevant in young animals compared to medium aged or older ones (Martinez-Canabal et al., 2013). Intriguingly, testing mice using a visual platform before subjecting them to hidden version within the same pool consistently attenuated the acquisition phenotype (Rola et al., 2004; Meshi et al., 2006; Zhang et al., 2008).

The fact that the acquisition phenotype appeared only using setups with a high number of landmarks arranged in a complex spatial configuration and when testing mice instead of rats suggests that adult neurogenesis only gets involved when information processing capacity in the DG is sufficiently stressed. Thus, whether differences between animals having normal levels of neurogenesis and those lacking the new neurons reach significance might also depend on the specific task demands. According to the least-effort principle, animals tested in a setup allowing to find the hidden goal without the need to separate a high number of contexts and thus stressing hippocampal networks will never show the expected phenotype.

While probe trial performance measures the final outcome of a preceding acquisition period, task acquisition also reflects a species' general spatial learning abilities, aspects related to adult neurogenesis being only a part of them. Consequently, given that rats have considerable higher cognitive abilities than mice, a task that is difficult for mice likely is less difficult for rats. The availability of distant visual cues represents an essential aspect of water maze setups. Most authors prefer a setup where animals can see only a small number of cues, either as posters or high-contrast patterns hanging on the walls of an otherwise empty, white painted room or being attached to a curtain around the pool. While such a setup is essential for studies addressing the relation of behavior or neuronal activity to the rotation, deletion, or addition of specific landmarks, it still represents an extremely reductionistic environment compared to a naturalistic situation where navigational abilities originally evolved. That issue gains specific importance in the context of adult neurogenesis, where the question is how the new neurons allow upholding efficient information processing when the brain has to deal with a high load of sensory information in a high number of similar, but still distinct contexts. Considering the essential types of information processing in the DG and CA3, theoretical models predicted a critical role for adult neurogenesis in learning tasks that specifically stress an animals' ability to discriminate spatially complex and highly similar contexts (Appleby and Wiskott, 2009; Appleby et al., 2011).

Introducing the animals at multiple and varying starting points promotes the formation of an allocentric cognitive map and it was shown that a lack of new hippocampal neurons selectively affects water maze performance relying on allocentric but not egocentric strategies (Dupret et al., 2008). It was also found that adult neurogenesis gets involved when spatial knowledge from multiple egocentric, route-based experiences have to be combined into an allocentric representation (Garthe et al., 2009). Therefore, using varying starting positions over multiple trials represents an essential prerequisite to get the new neurons involved and to obtain the acquisition phenotype. As an equally important co-factor, the experimental setup has to ensure the availability of a high number of potential landmarks resulting in higher task demands. Regarding the acquisition phenotype, exposing the tested animals to a visual platform test preceding the hidden goal phase was found in several studies to be detrimental. Although the exact mechanism explaining that findings is still missing, it is reasonable to assume that mice encoded the platform as part of certain panoramic views. Consequently, such animals would never need an allocentric cognitive map to find the hidden goal.

After moving the platform to a new position, efficient navigation to the hidden goal has to be relearned and the cognitive map underlying navigation has to be updated (Figure 3). Although the general task and spatial context remains unchanged, the relearning process represents a new acquisition because the behavioral endpoint of the task changed. The reversal phenotype explicitly refers to the ability of the DG network to separate contexts and a lack of new hippocampal neurons was predicted to cause exactly the phenotype observed (Wiskott et al., 2006).

Summarizing what can be learned from previous studies regarding setups and protocols revealing a function of adult neurogenesis, an experiment aimed to show all three phenotypes reliably would have to test mice in a pool without curtains but a normally equipped room, trained for multiple days with a constant platform but varying starting positions, a probe trial after the end of the acquisition period, finally followed by another acquisition where the goal has been moved. While the following sections discuss the specific relevance of assessing the allocentric aspects of spatial water maze learning, the issue of separating contexts and how this relates to the reversal phenotype is discussed further below.

## Classical water maze parameters alone do not allow assessing the hippocampus-specific aspects of spatial learning

Animals with hippocampal lesions cannot form an allocentric cognitive map (Eichenbaum et al., 1990). Because adult neurogenesis is found in the hippocampus and thus presumably affects spatial learning behavior that is linked to allocentric representations, the use of parameters assessing the allocentric aspects more specifically represents a key issue for characterizing the role of new hippocampal neurons in water maze learning. Classical parameters widely used to quantify spatial learning performance in the water maze such as latency to reach the platform and swim path length address the animals' overall learning performance in the task. Taking into account the differences in overall performance between species along with the role of multiple brain systems in spatial learning aforementioned, these parameters do not allow assigning differences in learning performance specifically to changes in the functional contribution of the hippocampus. For example, animals can show a longer latency, but still an almost direct approach toward the hidden goal. However, there might be differences in the respective swimming speed, number and duration of floating periods, general motivational issues and alike, all factors potentially explaining the differences in latency and path length observed.

Albeit to a lesser extent, the same problem holds true for path length. Although this parameter is more closely linked to the spatial aspects of water maze performance, it is still susceptible to subtle motoric, sensory, or motivational problems as well as deficits in route selection or general executive control. Consequently, assessing latency and path length alone is not sufficient to conclude that a given phenotype in the water maze can be explained by changes in hippocampal function. Nonetheless, many studies using the water maze task actually do so.

While latency and path length generally reflect how spatial memory is acquired and which performance levels are reached during and at the end of acquisition, the focus of parameters assessing probe trial performance lies on the question whether animals developed a clear preference for the goal location and thus whether they formed a cognitive representation allowing efficient navigation. Usually, probe trial performance is addressed by calculating the relative amount of time spent in the correct goal quadrant. This measurement, however, cannot be considered to be highly specific, since the probability is high that an animal enters the goal quadrant that is covering 25% of the pool surface just by chance. On top of this, the probability of an animal to be found at a given location is not evenly distributed for the entire pool: the position at which the animal is introduced to the pool relative to the goal determines certain areas that invariably must be crossed to reach the hidden goal.

Counting how often an animal crosses the former goal position solves this problem and provides more information on the spatial accuracy with which the platform position has been encoded. Because a high number of goal crossings is indicating a successful encoding of the exact goal position that in turn is presumed to be dependent on efficient pattern separation in the DG, that parameter appears to be a good candidate for assessing the functional relevance of adult neurogenesis.

It is obvious, that the arguments raised against using only latency and path length as parameters to proof hippocampus-specific changes in spatial learning also hold true when addressing the functional relevance of adult neurogenesis. Thus, among the “classical” parameters, only the number of former goal crossings in a probe trial as an estimate of how specific the goal location has been encoded can serve as a measurement that reasonably reflects aspects of hippocampal function assumed to be related to adult neurogenesis.

## Analysis of search strategies used in the water maze task

A particularly useful approach to assess the underlying representations that facilitate an observed behavior has been identified in analyzing the overall search patterns or strategies of animals attempting to find a hidden goal in the water maze. Most importantly, this approach allows differentiating hippocampus-dependent allocentric and hippocampus-independent egocentric search strategies. Analyzing the search strategies based on strictly defined numerical parameters was introduced by Wolfer et al. (2001).

**Figure 4:**
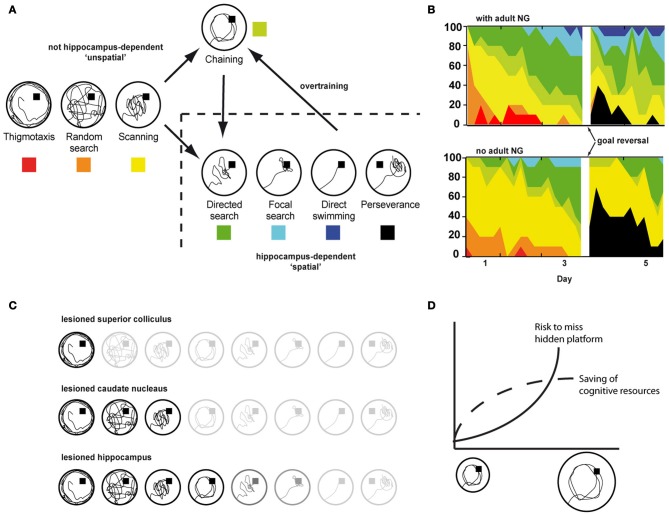
**(A)** Search strategies used by rodents to locate the hidden platform in the water maze. **(B)** Progression toward hippocampus-dependent search strategies with (upper panel) and without adult neurogenesis (lower panel). The experiment included two acquisition phases before and after a platform reversal (Garthe et al., 2009). **(C)** Effects of selective lesions to different brain structures on search strategies in the water maze. Blurred items represent strategies not shown following the indicated lesion. **(D)** Chaining as an instantiation of the least-effort-principle in small but not in large water maze arenas.

Laboratory rodents show a stereotypic sequence of search patterns for finding the hidden platform in the water maze task (Figure 4A). More efficient strategies show clear preferences for specific places allowing to reach the hidden goal using either directed route-based strategies or even straight approaches to the goal or its vicinity. While directed, route-based learning represents the increasing integration of egocentric route-knowledge into an allocentric representation, straight approaches toward the platform from any possible starting point explicitly rely on such a cognitive map and thus, the hippocampus gets increasingly involved as the relative contribution of allocentric knowledge increases.

The increasing contribution of allocentric knowledge is represented by an increase in the accuracy with which an animal is able to approach a hidden goal directly (Figures 3, 4A). While a lower allocentric contribution is indicated by an only rough estimate of goal direction (i.e., directed search) resulting in directed but not precise search strategies, later stages become increasingly precise and are characterized by direct goal approaches from any possible starting position (i.e., focal and direct search). Lesions of the hippocampus effectively prevent such allocentric strategies to occur (Eichenbaum et al., 1990), Figure 4C.

It is important to point out that the categories used to classify a given trial as a certain strategy are merely descriptive, i.e., swim paths with similar general characteristics get assigned to the same strategy. The characteristics defining a certain strategy are described by a maximum of two numerical parameters such as pool coverage, time spent in zone, or agglomeration. Each strategy relies on the contributions of several brain regions, the respective functional contributions allowing search patterns with unique and more common features that are shared with other strategies. Therefore, a given strategy does not represent the activity of a single functional system of the brain. However, lesions to specific brain structures prevent the associated function to contribute to all strategies it is relevant. For example, lesioning the superior colliculus effectively prevents an animal to turn toward any kind of cue and thus this will effect all strategies following random swimming (Dräger and Hubel, 1975; Ellard and Goodale, 1988), Figure 4C. Animals with lesions to the posterior cingulate cortex show an strongly impaired route navigation (Sutherland and Hoesing, 1993). Such animals can show taxis behaviors and even travel along know routes, but will never develop a preference for the hidden platform that represents the reward. Of specific importance for addressing the functional relevance of adult neurogenesis, animals without a working hippocampus cannot form an allocentric cognitive map and thus have to rely on egocentric strategies. Consequently, route-based and even directed search patterns can be found, but as the strategies become more straight, precise and thus allocentric, animals with hippocampal lesions fail (Morris et al., 1982; Eichenbaum et al., 1990).

However, egocentric route-based strategies can be highly efficient for solving the task and thus, decreasing latencies or path lengths can be found in animals with hippocampal lesions. For example Schallert et al. showed that using a specific and sophisticated protocol allows to find the hidden platform exclusively by means of an highly egocentric strategy (Day and Schallert, 1996). Such egocentric strategies can be rather direct, causing short latencies that mimic the efficiency of allocentric strategies but missing their flexibility.

Using varying starting points promoting the use of allocentric strategies can help to prevent this problem. In line with this, it was demonstrated that a protocol promoting the development of egocentric strategies did not cause the acquisition phenotype observed after suppression of adult neurogenesis when using varying starting points (Dupret et al., 2008). However, promoting the use of allocentric strategies by using multiple starting points is not sufficient to exclude any role of egocentric search strategies. Eichenbaum et al. demonstrated that even mice with hippocampal lesions were able to learn the water maze as indicated by decreasing latency times and path lengths (Eichenbaum et al., 1990). It turned out that the animals just learned the goal's correct distance to the pool wall and thus located the hidden platform effectively by swimming a circular path parallel and at the right distance to the wall. This can result in latency times nearly as low as found for mice using supposedly allocentric strategies. Analysis of several thousand trials from our own lab revealed that such a chaining strategy accounts for around 10–15% of trials that—regarding their respective latency values—would have been considered to be representing an allocentric search strategy. According to the least-effort principle using the chaining strategy allows to save cognitive resources while keeping the risk of missing the hidden goal low. With increasing pool size the risk to accidentally miss the platform increases and using an allocentric strategy becomes the preferred option (Figure 4D).

Therefore, combining a protocol promoting the formation of allocentric representations with a strategy analysis that allows excluding effective but egocentric strategies substantially increases the ability to assess specifically those aspects of water maze learning that involve information processing in the hippocampus and thus can be supposed to be related to adult neurogenesis.

Also in some studies strategies were identified by human raters, classification should be done based on clear defined numerical parameters with a maximum of two parameters per strategy. Besides being faster, such an algorithm-based classification avoids any bias-problems of human raters. Parameters used for classification usually relate to the amount of time spent in a specific area of the pool, and how effective the search is confined closely nearby or directed toward the hidden platform. The parameters that are used to automatically identify strategies are only descriptive of certain key-features of patterns in the stereotypic sequence. Considering our own data on several thousand trials, the algorithm-based classification turned out to be highly reliable, as only 5–15% of the swim patterns had to be verified or re-checked by human raters.

An important issue especially in the context of studies using genetically or otherwise modified or treated animals to ablate (or boost) adult neurogenesis is, that the analysis of search strategies can reveal changes in spatial learning that—according to classical water maze parameters—result in overall changed performance levels but in fact are not related to the new neurons. On the other hand, we have demonstrated that ablation of adult neurogenesis results in highly specific functional deficits exclusively in hippocampus-dependent strategies, while other aspects of spatial learning remained unaffected (Figure 4B). Therefore, analyzing the strategies helps to increase the specificity in addressing the functional effects of new hippocampal neurons and to rule out both unwanted and underestimated side effects not attributable to adult neurogenesis. As an example, convolution analysis revealed that failing to use hippocampus-dependent strategies following ablation of adult neurogenesis was not due to a generally impairment in “strategy switching” (and thus executive control) but due to selective deficits in applying spatially/contextually specific and thus hippocampus-dependent strategies (Garthe et al., 2009).

Both the acquisition and reversal phenotype observed after suppression of adult neurogenesis are characterized by higher latency times and swim path lengths. However, the effects appeared to be rather transient, i.e., even animals with reduced numbers of new neurons finally reached comparable performance levels but showed significant impairments during acquisition (Zhang et al., 2008; Garthe et al., 2009). Analyzing the search strategies allows estimating the effects of adult neurogenesis on how effective egocentric knowledge is combined into an allocentric cognitive map and how the allocentric contribution to water maze performance increases over multiple trials.

Another advanced parameter that was introduced in a study testing human subjects in a virtual water maze task is the initial heading error (Woolley et al., 2010). This parameter primarily addresses a subjects' ability to choose a direct and efficient route to the goal, right from the starting point. Any aberration from the direct start-goal connection increases the initial heading error until the overall distance traveled equals the lengths of the direct start-goal connection. While initial heading also relies on a successfully acquired cognitive map, there is some evidence from human studies that the precision of initial approaches reflects the ability to select direct routes toward the goal, and that these process is primarily mediated by parahippocampal areas (Wolbers and Hegarty, 2010). Thus, small initial heading errors provide implicit evidence for effective information processing in the hippocampus, resulting in the presence of a precise allocentric cognitive map.

To conclude, as compared to latency and path length, assessing search strategies, number of goal crossings, and initial heading error address the formation of an allocentric cognitive map and thus implicitly efficient information processing in the DG more specifically. However, considering the specificity of assumptions and predictions made by the theoretical models, especially regarding the orthogonalization of activity patterns in CA3, it becomes clear that the protocols and parameters generally used to address the functional relevance of adult neurogenesis are still rather limited. Referring again to the findings by McNaughton et al., these limitations appear to be mainly rooted in conceptual rather than technical issues. Therefore, we hypothesize that learning the water maze especially asks for an efficient encoding of the invariant features of the task that allow to define specific contexts. Re-acquisition after moving the hidden platform and the associated reversal phenotype are intimately related to the concept of contexts in water maze learning that is discussed in the following section.

## The role of contexts in water maze learning and implications for a functional role of adult neurogenesis

Living organisms learn to adapt their behavior to specific contexts in order to master the challenges posed by that situations and to make predictions for the future. Moving around freely in a world that essentially is representing an ever-changing environment with only a few stable “rules” raises significant demands on an animal's ability to recognize already known contexts, to express a specific behavior associated with a known context, and to re-engage in learning new associations of contexts and behaviors.

The principle goal of the water maze task is to navigate effectively toward a hidden platform within the pool. Contexts are provided by unique configurations of distant visual landmarks outside the pool that are available in the testing room and allow associating specific areas in the pool with a specific moving direction that changes the animal's relative position to the goal (Figures 2, 3). Trying to find the hidden platform over a number of trials allows accumulating knowledge about the invariant spatial relations of cues and thus of specific contexts. In essence, it is the flexible use of known contexts that facilitates the acquisition of egocentric route-knowledge.

A single water maze trial represents not only a swim path leading to the hidden platform but also a sequence of episodes experienced within a short time window and in specific contexts. Because under the rather artificial testing situation pool size, as well as number and configuration of landmarks around the water maze arena remain constant, specific contexts are experienced repetitively within or across trials. Therefore, certain spatial contexts gain a predictive value and thus specific relevance when they are recognized while the animal is trying to obtain a reward, i.e., reaching the hidden platform. Consequently, successful differentiation of contexts and combining those contexts into an allocentric cognitive map allows the use of directed and efficient search strategies (Figure 3).

A specific context is also defined by the actual goal position as a key element of the task and thus does also rely on non-spatial, task-related parameters. The fact that certain episodes (i.e., taken from the home cage into the pool or finding the goal in a certain quadrant) are repetitively experienced and will be conceived as “stable.” These circumstances are likely an important factor in defining “context” and at least in theory have to be differentiated from others to master a given task successfully.

When the goal and thus the reward has been moved to a new position following a goal-reversal, the general context fundamentally changes (Figure 3). Already known places that have been associated with the previous goal position now appear in the new context and their previous predictive value is not longer valid. Effective navigation to the new goal position demands forming new associations of places and moving directions. Given that the same set of visual cues is still present and the geometrical configuration remained unchanged, the new associations necessarily shows a considerable overlap with those learned in the context of the first goal position. Thus, to keep overall task performance efficient successful differentiation of contexts defined by changing goal positions is essential.

Considering the previously mentioned reversal phenotype as well as predictions from theoretical models, we propose that the addition of new neurons to the DG allows adapting rapidly to the new context. Relating to the cognitive reserve hypothesis it can be presumed, that as overarching goals and “rules” more likely change over longer periods compared to the rather short-term and simplistic laboratory behavioral tasks, the full gain-of-function by new hippocampal neurons likely become more apparent on longer timescales only. Therefore, to address the potential contribution of adult neurogenesis in situations where multiple goals that are associated to similar contexts have to be separated, one has to use an experimental water maze protocol that provides a single or multiple platform reversals.

The concept of contexts defined by the spatial and task-related invariants combined with the aforementioned relevance of overall task demands could help to explain the somewhat surprising results obtained in a study assessing the functional consequences of suppressing adult neurogenesis in another spatial learning task.

Testing mice in a radial arm maze revealed that an ablation of adult neurogenesis can led to an overall increased learning performance (Saxe et al., 2007). This seemingly paradoxical result was interpreted referring to the high excitability of the newborn neurons in the hippocampus and it was proposed that the new cells facilitate the encoding of the underlying task rules (Aimone et al., 2009). According to this hypothesis, immature new neurons form broader and less differentiated representations for contexts experienced closely related in time, allowing the generalization of “rules.” Testing animals in the same paradigm but using considerably different protocols, other authors did not find the improvement previously reported (Clelland et al., 2009). In their original report Saxe et al. however, had placed specific emphasis on differentiating visits in adjacent arms, closely related in time. In that special case a lack of new plastic neurons would allow to treat each visit as a single event, resulting in an overall higher task performance.

In naturalistic contexts, however, the ability to identify and to remember contexts experienced repetitively, as well as actions associated with those contexts represents a critical factor for increasing an organism's learning performance. The encoding of spatial and temporal invariances of a given environment allows to minimize threads and to increase the chance for obtaining rewards. In short, it helps to invest available resources more efficiently by exploiting the predictive values of well-known contexts. In principle, this also applies to the water maze task, especially in setups providing a high number of cues and thus providing numerous spatially defined contexts on the pool surface that can be used for navigation.

Considering the fact that the hippocampus receives sensory information from all modalities, the situation encountered in the water maze pool can be seen as somewhat deprived as only visual information can be used as a reliable source for context-defining spatial information. Since mice usually rely on olfaction, in dry land environments the actual contribution of odors might be even higher compared to visual information. However, the concept of contexts presented above still holds true, because contexts can principally be (and in naturalistic environments probably are) defined by information of all available modalities.

## Conclusion

In conclusion, the variety of setups, protocols, and parameters used to address the functional contribution of adult neurogenesis to spatial learning in the water maze task do not always reflect the specific prerequisites presumed to be necessary to get the new neurons involved. The significant mismatch between the inconsistent results from numerous water maze experiments and the rather homogenous theoretical concepts proposed for the functional relevance of adult neurogenesis is taking the classical ways to set up and analyze a water maze experiment into question.

Considering the phenotypes repeatedly found in experimental studies as well as ideas offered by theoretical models of adult neurogenesis revealed important roles for the overall task demands and invariant contexts encountered during the task. It became clear that the successful assessment of the truly allocentric aspects of spatial learning in the water maze represents a key point to understand where, when, and how the new neurons contribute to hippocampal function.

The water maze still represents the gold standard for testing hippocampal function in rodents. As our understanding of how different functional brain systems cooperate with each other as well as with adult neurogenesis to improve spatial navigation, the setups, protocols, and parameters used must reflect that understanding. The proposed concept of contexts defined by both physical cues and rather abstract task-related aspects can provide a starting point for devising new approaches to address the functional relevance of adult hippocampal neurogenesis in spatial water maze learning.

### Conflict of interest statement

The authors declare that the research was conducted in the absence of any commercial or financial relationships that could be construed as a potential conflict of interest.
